# Ginkgetin Alleviates Inflammation, Oxidative Stress, and Apoptosis Induced by Hypoxia/Reoxygenation in H9C2 Cells via Caspase-3 Dependent Pathway

**DOI:** 10.1155/2020/1928410

**Published:** 2020-11-04

**Authors:** Xin Liu, Hong Bian, Qing-Li Dou, Xian-Wen Huang, Wu-Yuan Tao, Wen-Hua Liu, Na Li, Wen-Wu Zhang

**Affiliations:** ^1^Department of Emergency Medicine, The Baoan Hospital Affiliated with Southern Medical University, People's Hospital of Baoan District of Shenzhen, China; ^2^Department of Cardiothoracic Surgery, Southern University of Science and Technology Hospital, China

## Abstract

Ginkgetin, the extract of *Ginkgo biloba* leaves, has been reported to exert preventive and therapeutic effects on cardiovascular disease. However, little is known about its role in myocardial ischemia-reperfusion injury (MIRI). The present study aimed to unveil the function of ginkgetin in cardiomyocytes subjected to hypoxia/reoxygenation (H/R) injury. Cell Counting Kit-8 (CCK-8) was employed to evaluate the impact of ginkgetin on cell viability in the absence or presence of H/R. Proinflammatory cytokines and malondialdehyde (MDA), reactive oxygen species (SOD), and lactate dehydrogenase (LDH) were determined via corresponding kits. In addition, flow cytometry was performed to detect apoptotic level. Western blot analysis was utilized to estimate caspase-3 and cytochrome C. Ginkgetin had no significant effect on cell viability; however, it could enhance viability of H9C2 cells exposed to H/R. Inflammation and oxidative stress induced by H/R injury were relieved via pretreatment with ginkgetin. Preconditioning of ginkgetin also decreased apoptotic rate and the protein levels of caspase-3, cytochrome C under H/R condition. Furthermore, 2-HBA, an inducer of caspase-3, was used for the activation of caspase-3 signaling pathway. It was found that induction of caspase-3 eliminated the protective effect of ginkgetin on H9C2 cells exposed to H/R. These results indicated that ginkgetin attenuated inflammation, oxidative stress, and apoptosis. These protective roles of ginkgetin may attribute to caspase-3 dependent pathway.

## 1. Introduction

Myocardial ischemia and hypoxia triggered by coronary circulation alterations are considered as the cornerstones of ischemic heart disease, which pose serious threats to human health [1]. Ischemic heart disease is often accompanied by myocardial ischemia-reperfusion injury (MIRI), the generated oxygen free radicals and apoptosis of which are the pivotal mechanisms of MIRI. Researches have illustrated that the apoptosis of myocardial cells induced by MIRI is closely associated with oxygen free radicals, calcium overload, mitochondrial damage, and heat shock protein [2–4], which is an important way to affect cardiac function [5].

MIRI is still a major clinical challenge; hence, many scholars are committed to discovering effective therapies to alleviate MIRI [6]. Numerous studies have found that pharmacological preconditioning is an effective approach for myocardial protection, with broad research prospects, which can enhance the body's ability to tolerate ischemia reperfusion, shrink the area of myocardial injury as well as improve prognosis [7–9].

The extract of *Ginkgo biloba* leaves has been widely used for the prevention and treatment of cardiovascular disease [10]. Ginkgetin, a biflavonoid from *Ginkgo biloba* leaves, manifests strong neuroprotection for SH-SY5Y and PC12 cells against cytotoxic insults induced by oxidative stress or amyloid beta [11]. It can also mitigate autophagy and apoptosis caused by cerebral ischemia/reperfusion (I/R) through inhibiting the NF-*κ*B/p53 signaling pathway [12]. Ginkgetin protects neurons against I/R-induced inflammation in rats via inhibiting the TLR4/NF-*κ*B pathway [13]. Moreover, I/R-induced apoptosis could be initiated by the release of caspase-dependent cytochrome C in cardiomyocyte injury model [14]. However, the role of ginkgetin in MIRI is rarely studied. In this work, we established hypoxia/reoxygenation (H/R) injury model to mimic MIRI and investigated the effects of ginkgetin on H/R injury from the perspectives of oxidative stress and apoptosis, among which caspases were further explored, intending to provide experimental foundation for application of ginkgetin.

## 2. Materials and Methods

### 2.1. Reagents and Antibodies

Ginkgetin (CAS No.: 481-46-9) and 2-hydroxy-benzylidene (2-HBA; CAS No.: 131359-24-5) were purchased from MedChemExpress (Shanghai, China). MTT was obtained from Sigma-Aldrich (St. Louis, MO, USA). Radio immunoprecipitation assay (RIPA) lysis buffer (P0013K) and BCA Protein Assay Kit (P0011) were obtained Beyotime Institute of Biotechnology (Shanghai, China). The anti-Bcl-2 (sc-7382) and anti-Bax (sc-7480) antibodies were purchased from Santa Cruz Biotechnology (CA, USA). The anti-Nox 2 (19013-1-AP), anti-Nox 4 (14347-1-AP), anti-GAPDH (10494-1-AP), and anti-cytochrome C (10993-1-AP) antibodies were obtained from Proteintech Group (Chicago, IL, USA). The anti-NF-*κ*B (ab194726), anti-pro-caspase-9 (ab184786), anti-caspase-9 (ab52298), anti-caspase-3 (ab13847), and anti-pro-caspase-3 (ab184787) antibodies were purchased from Abcam (Cambridge, UK). HRP-conjugated Affinipure Goat Anti-Mouse IgG (SA00001-1) and HRP-conjugated Affinipure Goat Anti-Rabbit IgG (SA00001-2) were obtained from Proteintech Group (Chicago, IL, USA).

### 2.2. Cell Culture and H/R Model

Rat H9C2 cells were purchased from Cell Bank of Chinese Academy of Sciences (Shanghai, China) and cultured in Dulbecco's modified Eagle's medium (Invitrogen, Carlsbad, CA) containing 10% fetal bovine serum (Hyclone, UT, USA) under a humidified atmosphere of 95% air and 5% CO_2_ at 37°C. Cells were treated with ginkgetin (1, 5, and 10 *μ*M) or vehicle (DMSO) at 70-80% confluence for 4 h prior to H/R. To establish the H/R model, H9C2 cells were maintained in serum and glucose-free DMEM under an atmosphere of 95% N_2_ and 5% CO_2_ at 37°C for 6 h followed by reoxygenation for 10 h with fresh culture medium (95% air and 5% CO_2_).

### 2.3. Cell Viability Assay

H9C2 cells were planted into a 6-well plate at a density of 1 × 10^4^ cells/well. After corresponding treatments, cells were incubated with 20 *μ*l MTT (0.5 mg/ml) for 4 h at 37°C in the dark. Next, 200 *μ*l DMSO was added into each well to dissolve the formazan crystals. The optical density (OD) was recorded on a BioTek microplate reader (BioTek, Richmond, VA, USA) at 490 nm. The results were represented as the relative percentage of the control group.

### 2.4. Measurements of LDH Activity, SOD Activity and MDA Content

The activities of LDH and SOD as well as the content of MDA were evaluated according to the manufacturer's protocol (Solarbio Science & Technology Co., Ltd. Beijing, China). In brief, medium was discarded, cells were collected into tubes, extraction reagent was added at a ratio of 1 ml reagent/5 × 10^7^ cells, and then, the mixture was centrifuged (8000 g) for 10 min at 4°C. For detection of MDA content, with addition of other reagents, the mixture was stored at 100°C for 60 min and centrifuged at 10000 g for 10 min after cooling. The absorbance value of each well was measured at 450 nm, 532 nm, and 600 nm. For LDH activity, corresponding reagents of LDH assay kit were supplemented, after which the mixture was incorporated thoroughly, placed at room temperature for 3 min, and then, the absorbance value was measured at 450 nm. To detect SOD activity, the mixture was placed in a water bath at 37°C for 30 min for measuring the absorbance value at 560 nm.

### 2.5. Measurements of Inflammation Cytokines and Cytochrome C

TNF-*α*, IL-6, IL-1*β*, HMGB1, and cytochrome C were estimated using Assay Kits (Jiancheng Bioengineering Institute, Nanjing, China) according to the manufacturer's protocol, respectively. Triplicate wells were set up for each group. Results were assessed from three independent experiments.

### 2.6. Western Blot Analysis

H9C2 cells were washed with PBS three times. After PBS was removed completely, RIPA lysis buffer was added, and then, cells were scraped and centrifuged at 10,000 g at 4°C for 15 min. Following the collection of the supernatant, protein concentrations were determined by using the BCA Protein Assay Kit. Proteins were separated by 10% SDS-PAGE gels and subsequently transferred onto the PVDF membranes (Millipore, MA, USA). The membranes were blocked with 5% nonfat milk at room temperature for 2 h, followed by incubation with the primary antibodies overnight at 4°C. Subsequently, membranes were conjugated with secondary antibodies at room temperature for 1 h. Protein quantity was analyzed using the Image J software (National Institutes of Health, Bethesda, MD, USA).

### 2.7. Flow Cytometry Analysis

The flow cytometry analysis was employed to detect apoptotic cells. H9C2 cells were pretreated with ginkgetin or 2-HBA and cultured in the absence or presence of H/R challenge. Apoptotic cells were examined by Annexin V-FITC Apoptosis Detection Kit (Sigma-Aldrich, St. Louis, MO, USA) according to the manufacturer's instruction. Briefly, after washing with PBS, cells were collected and incubated with binding buffer at 4°C for 10 min at room temperature in the dark. Next, cells were analyzed by flow cytometry immediately. Based on the flow cytometry scatter diagrams, Q1 quadrant represented necrotic cells and Q4 quadrant represented living cells. The total apoptosis rate was calculated as the sum of the Q2 (late apoptotic cells) and Q3 quadrant (early apoptotic cells).

### 2.8. Statistical Analysis

Data were presented as the mean ± SD. Each experiment was repeated three times. One-way ANOVA was performed for multiple-group analysis, and Student's *t*-test was exploited to compare differences between two groups by GraphPad Prism 6.0. A value of *P* < 0.05 was considered statistically significant.

## 3. Results

### 3.1. Ginkgetin Relieves Inflammation of H9C2 Cells Triggered by H/R

**Figure 1:**
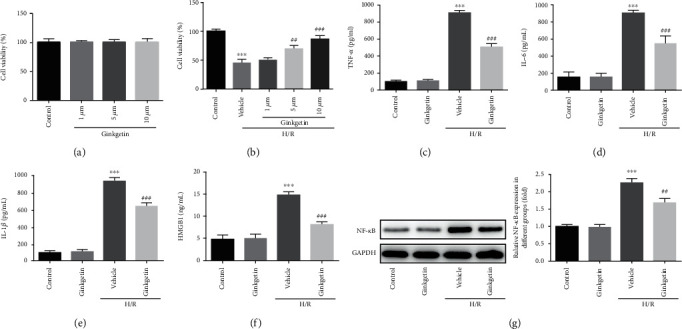
Ginkgetin relieves inflammation triggered by H/R in H9C2 cells. (a) CCK-8 assay was employed to estimate viability of H9C2 cells pretreated with ginkgetin. (b) H9C2 cells were pretreated with ginkgetin and exposed to H/R. Cell viability was estimated via CCK-8. C-F, TNF-*α*, IL-6, IL-1*β*, and HMGB1 were determined via assay kits. (g) NF-*κ*B was detected via western blot analysis. ^∗∗∗^*P* < 0.001 versus control group; ^##^P < 0.01, ^###^P < 0.001 versus vehicle group.

In order to assess the impact of ginkgetin on H9C2 cell viability, cells were pretreated with different concentrations of ginkgetin. The result of CCK-8 assay manifested that ginkgetin had no effect on cell viability at the dose of 1, 5, and 10 *μ*M (Figure 1(a)). Then, we estimated viability of H9C2 cells exposed to H/R; it was observed that H/R apparently decreased cell viability; however, pretreating cells with ginkgetin could elevate viability in a dose-dependent manner (Figure 1(b)). Hence, 10 *μ*M ginkgetin was applied in follow-up experiments. In addition, inflammatory cytokines of H9C2 cells exposed to H/R were significantly increased, while ginkgetin preconditioning greatly ameliorated inflammation through reducing the secretion of TNF-*α*, IL-6, IL-1*β*, and HMGB1 (Figure 1(c)–1(f)). NF-*κ*B involves in the response of cells to external stimuli, playing a key role in cellular inflammatory response. H/R drastically promoted the protein level of NF-*κ*B in H9C2 cells, which was inhibited under pretreatment with ginkgetin (Figure 1(g)). These results illustrated that ginkgetin preconditioning effectively ameliorated H/R-induced inflammation.

### 3.2. Ginkgetin Alleviates H/R-Induced Oxidative Stress and Apoptosis of H9C2 Cells

**Figure 2:**
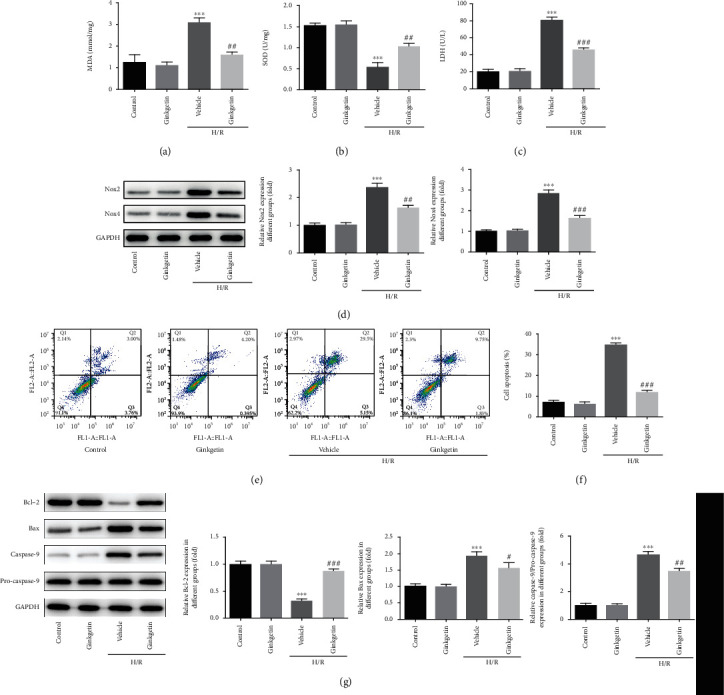
Ginkgetin alleviates H/R-induced oxidative stress and apoptosis of H9C2 cells. (a–c) MDA, SOD, and LDH were detected using assay kits. (d) Western blot analysis was conducted to assess the protein levels of Nox2 and Nox4. (e, f) Flow cytometry analysis was employed to screen apoptotic rate. (g) Bcl-2, Bax, caspase-9, and pro-caspase-9 were estimated by western blot analysis. ^∗∗∗^*P* < 0.001 versus control group; ^#^P < 0.05, ^##^P < 0.01, ^###^P < 0.001versus vehicle group.

Oxidative stress and apoptosis of H9C2 cells were examined in subsequent experiments. It was identified that MDA content in H9C2 cells subjected to H/R was markedly upregulated but reduced markedly when cells were pretreated with ginkgetin (Figure 2(a)). SOD activity was downregulated in H9C2 cells under H/R condition; however, ginkgetin partly restored SOD activity (Figure 2(b)). Ginkgetin also decreased H/R-triggered LDH release of H9C2 cells (Figure 2(c)). Nox2 and Nox4 belong to NADPH oxidase; the main function of them is to produce ROS [15, 16]. Nox2 and Nox4 were both enhanced after cells challenged with H/R, whereas ginkgetin preconditioning limited the elevation of Nox2 and Nox4 (Figure 2(d)). Furthermore, we conducted flow cytometry to evaluate apoptotic rate. Extensive apoptotic cells were observed after cells were subjected to H/R, whereas ginkgetin successfully reduced the number of apoptotic cells (Figure 2(e)). Determinations of apoptosis-related proteins Bcl-2, Bax, and caspase-9 validated the antiapoptotic effect of ginkgetin (Figure 2(f)). Collectively, ginkgetin could alleviate H/R-induced oxidative stress and apoptosis.

### 3.3. Ginkgetin Attenuates Inflammation Induced by H/R through Caspase-3 Dependent Pathway

**Figure 3:**
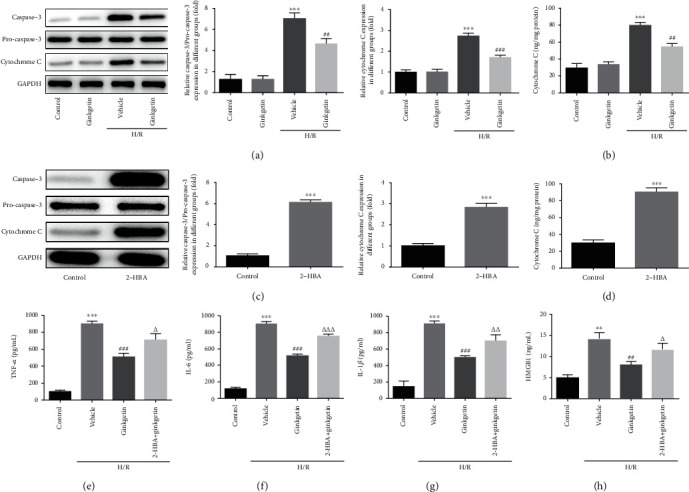
Ginkgetin attenuates inflammation induced by H/R through caspase-3 dependent pathway. (a) Cytochrome C, caspase-3, and pro-caspase-3 were estimated by western blot analysis. (b) Cytochrome C was evaluated using an assay kit. (c) H9C2 cells were treated with 0.6 *μ*M 2-HBA, and then, cytochrome C, caspase-3, and pro-caspase-3 were estimated via western blot analysis. (d) Assay kit was employed to determine the level of cytochrome C. (e–h) TNF-*α*, IL-6, IL-1*β*, and HMGB1 were determined using assay kits. ^∗∗∗^*P* < 0.001 versus control group; ^##^P < 0.01, ^###^P < 0.001versus vehicle group; ^△^P < 0.05, ^△△^P < 0.01, ^△△△^P < 0.001 versus ginkgetin group.

The Bcl-2 protein family determines the commitment of cells to apoptosis [17]. The mitochondrial apoptotic pathway is largely mediated through Bcl-2 family, which inhibits the mitochondrial release of cytochrome C [18]. Cytochrome C leakage can activate caspase-9, which in turn activates caspase-3 [19]. Notably, ginkgetin had no obvious influence on the expression of cytochrome C or caspase-3 in H9C2 cells under basal condition, while it significantly decreased levels of cytochrome C and caspase-3 in H9C2 cells exposed to H/R (Figure 3(a)). A similar result was testified via cytochrome C immunoassay kit (Figure 3(b)). In order to investigate whether anti-inflammatory effect of ginkgetin depends on caspase-3, 2-HBA, an activator of caspase-3, was exploited for gain-of-function studies. 2-HBA significantly promoted the expression of cytochrome C and caspase-3 (Figures 3(c) and 3(d)). Subsequently, TNF-*α*, IL-6, IL-1*β*, and HMGB1 were assessed again, which found that 2-HBA could partially abrogated the anti-inflammatory effect of ginkgetin (Figures 3(e)–3(h)). The detection of NF-*κ*B also validated the outcome (Figure 3(i)). Taken together, these results indicated that ginkgetin relieved inflammation triggered by H/R may, which depended on caspase-3.

### 3.4. Ginkgetin Ameliorates H/R-Triggered Oxidative Stress and Apoptosis via Caspase-3 Dependent Pathway

**Figure 4:**
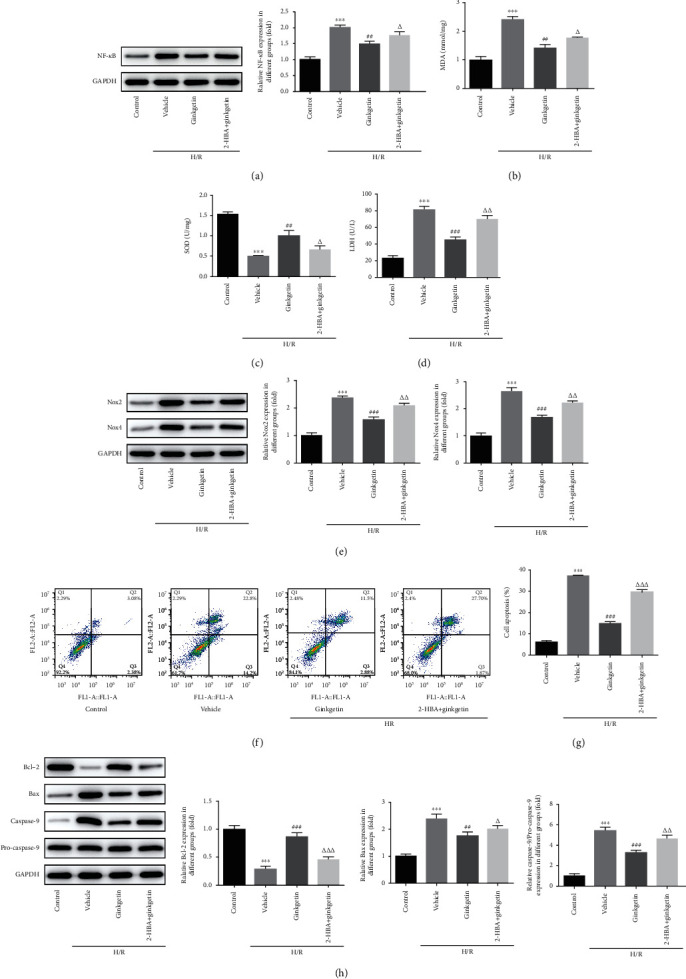
Ginkgetin ameliorates H/R-triggered oxidative stress and apoptosis via caspase-3 dependent pathway. (a) Western blot analysis was conducted to assess the protein expression of NF-*κ*B. (b–d) MDA, SOD, and LDH were detected using assay kits. (e) Levels of Nox2 and Nox4 were detected via western blot analysis. (f, g) Flow cytometry analysis was employed to screen apoptotic rate. (h) Bcl-2, Bax, caspase-9, and pro-caspase-9 were estimated by western blot analysis. ^∗∗∗^*P* < 0.001 versus control group; ^##^P < 0.01, ^###^P < 0.001 versus vehicle group; ^△^P < 0.05, ^△△^P < 0.01, ^△△△^P < 0.001 versus ginkgetin group.

To further explore the oxidative stress and apoptosis involved in ginkgetin-mediated caspase-3 pathway, relevant experiments were performed. Cotreatment of 2-HBA and ginkgetin enhanced MDA content and LDH activity in H9C2 cells, contrasted by ginkgetin preconditioning alone under H/R condition, whereas SOD exhibited an opposite trend with MDA and LDH (Figures 4(a)–4(c)). The expression of Nox2 and Nox4 in different groups also demonstrated the antioxidative capacity of ginkgetin was counteracted by 2-HBA (Figure 4(d)). Furthermore, a number of apoptotic cells detected via flow cytometry in cotreatment group were more than that in ginkgetin group (Figure 4(e)). Finally, Bcl-2, Bax, and caspase-9 were estimated using western blot analysis; results of which showed that 2-HBA reversed the antiapoptotic effect of ginkgetin (Figure 4(f)). Overall, these results displayed that 2-HBA could counteract the functions of ginkgetin towards oxidative stress and apoptosis under H/R condition.

## 4. Discussion

The basic physiological process of ischemic heart disease is myocardial ischemia. The main strategy in the clinical therapy of ischemic heart disease is restoring the blood perfusion to ischemic myocardium as soon as possible [20, 21]. However, blood reperfusion often leads to more severe injury to ischemic myocardium, also called MIRI [22]. It is generally believed that the mechanism of MIRI includes the outbreak of free radicals, mitochondrial damage, cell apoptosis, and inflammation, and that these injury factors are interrelated and often trigger or indirectly aggravate another injury factor.

Pharmacological preconditioning is one of the important approaches to relieve MIRI [23]. Previous researches have elucidated the anti-inflammatory and antioxidative properties of ginkgetin [24–27]. In addition, ginkgetin could attenuate cerebral I/R-induced injury [12, 13, 28], suggesting that ginkgetin can be a promising treatment for IRI.

In this study, H9C2 cells, subclone of the original clonal cell line derived from embryonic rat heart tissue [29], may not be representative of effects potentially seen in the intact myocardium; however, they are widely used for the establishment of H/R injury cell model to mimic MIRI [30, 31]. In this work, ginkgetin preconditioning hardly affected H9C2 cell viability; however, it enhanced the viability of cells under H/R condition. Existing reports have shown that I/R results in significant oxidative stress, further promoting cardiomyocytes death [32–34]. Herein, H/R also altered inflammatory factors and oxidative stress of H9C2 cells, which were alleviated effectively by pretreatment of ginkgetin. It is well-known that reperfusion after myocardial ischemia causes cardiomyocytes apoptosis [35, 36]. We analyzed apoptotic rate of H9C2 cells via flow cytometry and unmasked that ginkgetin could inhibit apoptosis of H/R-treated H9C2 cells. Moreover, ginkgetin preconditioning reduced the expression of caspase-3 and cytochrome C. To verify whether the myocardial protective effect of ginkgetin depended on caspase-3 signaling pathway, 2-HBA, an inducer of caspase-3, was employed. It was found that 2-HBA eliminated significantly the anti-inflammatory, antioxidative, and antiapoptotic effects of ginkgetin on H/R-injured H9C2 cells, implying that ginkgetin protected H9C2 cells against H/R injury through caspase-3 signaling pathway.

The signal transduction of apoptosis is divided into two basic pathways, exogenous pathway that is mediated by death receptors such as TNF-*α*, TRAIL, and FAS-L [37, 38], and another endogenous pathway regulated by the increased permeability of mitochondrial outer membrane [39]. This study focused on endogenous apoptosis pathway through investigating caspase-9, caspase-3, and cytochrome C. Members of the Bcl-2 family are located in mitochondria. They control mitochondrial permeability, cytochrome C release, and initiator caspase-9 activation, subsequently activating executor caspase-3 and exerting proapoptosis function [40, 41]. However, the regulatory mechanism of cytochrome C release and mitochondrial membrane permeability during apoptosis has not been fully elucidated. Future work will pay close attention to the regulatory signal of cytochrome C release through testifying the activities of caspases and mitochondrial membrane potential. In summary, our present study manifested that ginkgetin alleviated H/R-triggered inflammation, oxidative stress, and apoptosis of H9C2 cells via caspase-3 signal pathway.

## Data Availability

All the data in this study are available upon request.
